# Using Inequality Measures to Incorporate Environmental Justice into Regulatory Analyses

**DOI:** 10.3390/ijerph10094039

**Published:** 2013-08-30

**Authors:** Sam Harper, Eric Ruder, Henry A. Roman, Amelia Geggel, Onyemaechi Nweke, Devon Payne-Sturges, Jonathan I. Levy

**Affiliations:** 1 Department of Epidemiology, Biostatistics & Occupational Health, McGill University, Montreal, QC H3A 1A2, Canada; 2 Industrial Economics, Inc., Cambridge, MA 02140, USA; E-Mails: eruder@indecon.com (E.R.); hroman@indecon.com (H.A.R.); ageggel@indecon.com (A.G.); 3 Office of Environmental Justice, US Environmental Protection Agency, Washington, DC 20460, USA; E-Mail: onyemaechi.nweke@hhs.gov; 4 National Center for Environmental Research, US Environmental Protection Agency, Washington, DC 20460, USA; E-Mail: payne-sturges.devon@epa.gov; 5 Department of Environmental Health, Boston University School of Public Health, Boston, MA 02118, USA; E-Mail: jonlevy@bu.edu

**Keywords:** regulatory analysis, health inequalities, environmental justice

## Abstract

Formally evaluating how specific policy measures influence environmental justice is challenging, especially in the context of regulatory analyses in which quantitative comparisons are the norm. However, there is a large literature on developing and applying quantitative measures of health inequality in other settings, and these measures may be applicable to environmental regulatory analyses. In this paper, we provide information to assist policy decision makers in determining the viability of using measures of health inequality in the context of environmental regulatory analyses. We conclude that quantification of the distribution of inequalities in health outcomes across social groups of concern, considering both within-group and between-group comparisons, would be consistent with both the structure of regulatory analysis and the core definition of environmental justice. Appropriate application of inequality indicators requires thorough characterization of the baseline distribution of exposures and risks, leveraging data generally available within regulatory analyses. Multiple inequality indicators may be applicable to regulatory analyses, and the choice among indicators should be based on explicit value judgments regarding the dimensions of environmental justice of greatest interest.

## 1. Introduction

Regulatory analyses, which focus on quantifying the health and environmental benefits of alternative policy measures, are required for major environmental regulations in the United States and elsewhere. While regulatory analyses often include some discussion about environmental justice implications, they rarely engage in formal quantitative analyses to compare how alternative policy measures could differentially influence environmental justice. 

Whether it is viable to conduct such analyses depends on a number of factors, including whether “justice” is something that can be analyzed or quantified. As discussed elsewhere [1], it is important to have clarity about core concepts and language to answer this question, in part because there are a number of similar terms used in the context of studying inequalities in health or environmental exposures (e.g., difference, disparity, inequality, inequity, disproportionality, justice). The key conceptual distinction for our purpose is between the concept of inequality and that of inequity. 

Inequality is a relative (*i.e.*, relational) concept that contains both qualitative and quantitative elements [2]. Inequality plays a central role in the context of philosophical discussions of justice, but primarily as a qualitative concept that involves comparisons between a group of different objects, persons, processes or circumstances (e.g., the opportunity for well-being, equality before the law). Such comparisons may also be quantitative, in which case the concept of inequality may refer specifically to the measurement of differences in the distribution of goods such as income or wealth [3]. 

In contrast to the concept of inequality, the term “inequity” refers specifically to a subset of measured inequalities that are judged to be unfair or unjust. Judgments concerning inequity rely on social, political and ethical discourse about what a society believes is unfair, and are thus considerably more difficult to quantify [4,5]. Determining whether, or how much of, observed inequalities are inequitable requires consideration of important issues such as whether the inequality is avoidable, unfair, or remediable [6]. The quantification of inequality in health or exposure to environmental hazards or benefits is therefore necessary, but not sufficient, for determining whether or not such a distribution is indeed inequitable. 

The implication is that quantitative metrics can be used to measure inequality in health outcomes, paralleling the structure of the regulatory analyses, but that a determination of inequity or injustice would be beyond the scope of such analyses. In other words, the tools of regulatory analysis are not well suited for determining which inequalities are unjust and unfair, or whether the processes that led to the status quo situation occurred fairly and reasonably. Nevertheless, given the core definition of environmental justice used by the federal government in the United States [7], which identifies minority and low-income groups as the target populations, addressing environmental justice within regulatory analyses first requires an understanding of inequality in risks between defined population groups. For some applications, it may also be valuable to consider inequality in regulatory costs (e.g., if the cost of the regulation is passed through to consumers in a manner that disproportionately affects certain population groups), but we focus herein on health related to environmental exposures. 

The question is therefore whether between-group inequality in environmental health risk could be reasonably characterized within regulatory analyses and, if so, what the most logical approaches are for doing so. In this paper, we provide an overview intended to help environmental regulatory analysts understand how health inequality can be measured and how inequality measures can be applied in a new context. We first review the literature on income and health inequality indicators to determine the viability of quantitative measures of environmental health inequality within regulatory analyses. Given these insights, we propose four fundamental attributes of health inequality measures that should be explicitly evaluated before selecting an indicator. We then focus on health inequality measures that provide between-group comparisons consistent with environmental justice concepts, and we conclude by providing a logical approach by which policy decision makers could select among candidate indicators. 

## 2. Measuring Health Inequality

We are primarily concerned with characterizing the degree of inequality across social groups in defined health outcomes and how that inequality changes as a function of regulatory measures targeting environmental exposures. While this has not been done to date within regulatory analyses, similar questions have been addressed in the realm of income inequality through the development of numerous indicators with various attributes over the last century [3,8]. Indicators such as the Gini coefficient, Theil’s index, and the Atkinson index have been regularly applied, with rigorous debate about the strengths and weaknesses of alternate measures and their interpretations. Each of these indicators employs a slightly different construct, and decision-makers may prefer certain indicators over others, but most of them adhere to basic established principles that facilitate their interpretation. 

Income is different from health in some fundamental ways, raising the question of whether and how the income inequality indicator literature can be leveraged to inform understanding of health inequality. As has been argued elsewhere [9], mortality or disease risk is strictly bounded between 0 and 1, while income is effectively unbounded (and may even be negative); income can be directly transferred between individuals to remedy existing inequality, while risk cannot be transferred in this manner; and health risk is a multi-dimensional construct with a complex time component. More fundamentally, income is a “good” and health risk is a “bad,” so quantitative indicators of the distribution of income will be interpreted differently from the same indicators as applied to health risk. Moreover, dichotomous health states may be defined in either “positive” (absence of disease) or “negative” (presence of disease) terms. This has important consequences for the comparative analysis of health inequalities [10,11,12,13]. 

Although these are important observations, the differences are not as stark as they may appear [14]. For example, health policy choices fundamentally deal with distributions and tradeoffs of health risk factors, so risk can be redistributed across the population even if it cannot literally be handed from one person to another. Some attributes of health inequality also make it less challenging to characterize than income inequality [15]. For example, there are fewer unit conversion issues. In addition, mortality is easier to define than income across numerous countries, although measurement issues can be challenging for morbidity outcomes and risk factors. Health measures can also be characterized in a variety of ways, including as “goods” (e.g., life expectancy) or “bads” (e.g., mortality risk), though consistency in how health states are defined for bounded health variables is important for comparative purposes.

Given this, health inequality has been characterized in the peer-reviewed literature and in policy analyses for decades, and there are numerous examples of quantitative metrics of inequality being applied to health outcomes. Relatively simple summary metrics have been used to characterize health inequality, comparing levels of health across different pre-defined groups [15]. The Gini coefficient and Atkinson index have been used to characterize health inequality between countries [15,16], as well as to evaluate changes in inequality resulting from environmental policy measures [17,18,19]. Numerous publications have applied the Concentration Index [20,21] to characterize health inequality [22,23]. Thus, the question is not whether health inequality can be meaningfully characterized, but rather how an indicator of health inequality should be constructed in the context of regulatory analyses. 

First, because the goal of these measures would be to address environmental justice concerns, they should be able to provide comparisons between socioeconomic or racial/ethnic groups of concern. However, this does not necessarily imply that only between-group comparisons are germane. For example, if a pollutant displays significant spatial variability with local “hot spots,” it could be important to target the high-risk individuals within a minority or low-income population to improve environmental justice, rather than reducing risks uniformly across the minority or low-income population, even if the between-group differences are reduced identically. More generally, understanding whether differences in risk are more strongly driven by geography, demographics, or other factors (e.g., behaviors, co-exposures) is important in designing optimal interventions. 

Second, because regulatory analyses focus on characterizing health benefits/harms from regulatory measures, any indicators of environmental inequality should be based on the corresponding distribution of health outcomes. Or, if data are lacking to characterize the distribution of health benefits corresponding to a regulatory measure, indicators should be based on the distribution of exposures to health-relevant pollutants, determining how that distribution changes as a result of the regulatory measure. The general point is that the outcomes used in the inequality indicator should aim to be consistent with those outcomes used to characterize aggregate benefits within the regulatory analysis [1]. As a corollary, because of the interest in environmental justice and comparisons among defined population groups, inequality analysis should take account of differences in baseline disease rates or key effect modifiers across such groups. Incorporating differences in baseline disease rates is important not only for appropriate application of inequality indicators, but also for identifying high-risk populations. Similarly, the same incremental exposure change could have a greater effect on some individuals than others, and a number of these modifying factors could be socioeconomically or racially patterned [24]. 

Third, inequality measures themselves often have little meaning absent a context for interpretation, but are useful for comparative purposes. This aligns well with the structure of regulatory analyses, which involve comparing a defined set of policy options with the status quo or baseline, to determine the benefits of the regulation (often in comparison with the costs). Inequality measures will therefore be most meaningful when multiple policy options are under consideration and analyses consider the degree to which inequalities change as a result of the policy options. 

Finally, inequality measures will also be interpretable only when they take account of baseline inequality and are evaluated in conjunction with health benefits. To illustrate the importance of baseline values, suppose that two different low-income populations could be targeted for risk-reduction measures. The magnitude of risk reduction would be the same for both measures, but one group has elevated baseline health risks in comparison to higher-income populations, while the second group does not differ in its baseline health risks. Clearly, the option targeting the first population would be preferable from an environmental justice perspective, all else being equal, but this difference would be masked if the baseline distribution were not incorporated into the analysis. Including health benefits data is critical because, without measures of the magnitude of health benefits, inequality metrics could be used to argue for “leveling down,” in which environmental justice concerns could be met by increasing exposures among high-income or non-minority populations [25,26]. Whether within the inequality indicator itself or as a separate measure used in multi-attribute decision-making, the magnitude of health benefits must be considered at the same time as the distribution of health benefits.

In summary, health inequality has been characterized in the peer-reviewed literature and in policy contexts for decades. Approaches to characterize health inequality have ranged from simple summary measures to more complex statistical formulations, but there is a strong consensus in the literature that it is appropriate to develop and implement health inequality measures. Although such measures have had limited application in the context of environmental regulatory analysis, the prior applications in the health literature suggest inequality analyses are feasible for assessments of environmental justice. 

## 3. Key Attributes of Health Inequality Measures

While inequality measures can be described by a number of attributes, including adherence to various mathematical axioms common within the income inequality literature, we focus in this paper on four choices that we consider to be fundamental for developing interpretable measures of health inequality—reference points, scale, social group ordering, and explicit value judgments [27]. In this section, we introduce some well-established inequality indicators and consider their attributes with respect to these four choices. We note that some of these topics have been reviewed extensively in the literature [1,28,29,30,31,32,33], and we focus herein on information that would help environmental regulatory analysts understand the implications of choosing a specific inequality measure. 

### 3.1. Reference Point for Comparisons

Any inequality measure reflects a comparison between a reference group and other members of the population (or, in the case of between-group comparisons, members of another population group). For example, each individual might be considered relative to the average member of the population, where the degree of inequality is an aggregation of the differences between each individual and the average. As discussed elsewhere [2,33], this is a reasonably intuitive comparison that is common to many inequality measures. However, it does not directly capture some of the philosophical constructs relevant to inequality. For example, some philosophers consider the status of the worst-off to be most relevant for considering the degree of inequality [34].

One could also consider each individual relative to the best-off person or social group in society [35,36,37]. This has some theoretical appeal, as it reflects the idea that the best-off person or social group is at an attainable level that others could achieve with improvements to the physical and social environment [1,33]. However, there are potential issues, as attaining the well-being of the best-off person or group may not be a realistic goal. More practically, it may be difficult to characterize or quantify the level of health risk for the best-off person or group in a regulatory analysis [1], and there could be statistical instability if this group is relatively small in size [33]. A variant of this comparison would consider the best-off person whose condition is not anomalous, which may provide a more attainable goal but can be hard to define and quantify [2]. 

A third formulation involves comparing health risks of an individual or group to all those who are better off, rather than just the single best-off group or person. This provides a greater characterization of the full range of health risks across the population in relation to one another, and is less dependent on the experience of the best-off individual or group [1,33]. This may be appealing, since it reflects the logical idea that the number of people who are better or worse off than an individual should matter. However, it can only be incorporated within a subset of statistical indicators, because it requires a number of pairwise comparisons to be calculated. 

While these reference points are the most common by far, multiple variants could be considered. For example, various points along the distribution could be selected rather than the average (e.g., the median), although this is rarely done because of the challenges in constructing a single equation that could be clearly presented, as would be needed for interpretable inequality measures. For health risks (as opposed to positive health states), it could be argued that comparison with the worst-off or worst-off person/group whose condition is not anomalous would have value, but it is more typical to consider inequality in the context of the positive steps that could be taken to move individuals to a preferred state.

Regardless, it is important to recognize that each statistical formulation has an implicit or explicit reference group defined, and that the choice of reference group needs to be consistent with the priorities and beliefs of decision makers. It is also important to recognize that this choice has some significant implications. Consider a simple example, in which there are 4 people in the world, with initial health status of 10, 8, 4, and 2, respectively (on a scale from 1 to 10, where 10 is perfect health). Suppose that a policy measure would lead the distribution of health status to change to be 9, 9, 4, and 2—effectively, a one-unit transfer from the healthiest to the second-healthiest. If each individual is compared to the best off, the situation is unequivocally better with respect to inequality—the gaps have changed from (2, 6, 8) to (0, 5, 7), so each person is closer to the ideal. However, if each individual is compared to the average, it is no longer the case that the situation is unequivocally better with respect to inequality—if all differences were equally weighted, inequality would be unchanged. If the individual with health status of 10 is considered “anomalous”, then the policy measure would increase inequality by widening the gap between the two worst off (4, 2) and the second-best-off individual (9). The choice of reference group and form of statistical comparison should be consistent with how decision makers would perceive alternative scenarios. 

### 3.2. Relative *versus* Absolute Inequality

Another one of the fundamental questions for any inequality measure is whether it is capturing relative or absolute comparisons among the population [38]. Some measures are based on differences between groups or individuals and the reference point, while others are based on ratios or are constructed in a manner that is scale invariant. In other words, if health risks for all members of the population increased by a factor of two, measures based on absolute inequality would change, while measures based on relative inequality would not. Similarly, if health risks for all members of the population increased by an additive constant, measures based on absolute inequality would not change, while measures based on relative inequality would change.

Further complicating this issue is the fact that for all-or-none health states (e.g., presence or absence of disease) the magnitude of relative inequality will depend on whether one considers inequality in the presence or absence of disease [10,11,12,13,39,40]. For this reason, in comparing the magnitude of relative inequality between two counterfactual situations, decision-makers should be consistent in how the health state is defined.

Whether relative or absolute inequality measures are more appropriate for health inequality in the context of regulatory analysis is not immediately obvious. On the one hand, environmental regulatory analyses typically apply results from epidemiological studies that generally calculate and report uncertainty on a relative scale. It is appealing to have an inequality indicator be insensitive to these relative uncertainties [1]. In addition, if the inequality indicators are applied to environmental exposures, it is beneficial for the results not to depend on whether exposures are reported in parts per billion or parts per million. However, the dominant use of relative risks in the epidemiological literature may obscure important differences in baseline risk across different contexts, although the historical justifications for the use of relative risks (e.g., transportability across studies or environmental contexts) may also apply in the context of absolute risk differences [41]. Measures of absolute inequality are expressed in the same units as the health outcome or exposure under consideration, which can facilitate closer links between inequality and average health. Given concern about the amount of societal resources required to remedy an existing inequality, the absolute difference in health risk may be an important consideration. More fundamentally, measures of relative inequality and absolute inequality may sometimes produce conflicting findings with respect to how health inequalities change after a policy or intervention [27]. 

When making this decision, it is important to recognize that the inequality measure in a regulatory analysis is not being used in a vacuum, and does not need to both capture environmental justice and overall health issues. In other words, a situation in which health status in a 4-person world changed from (10, 10, 8, 8) to (5, 5, 4, 4) is a much worse situation all things considered, even if relative health inequality had not changed. An absolute inequality measure may most appropriately reflect the priorities and perspectives of decision makers, but it should not be selected solely because the amount of health risk matters as a separate decision parameter. However, some decision makers might determine that (10, 10, 8, 8) is a more unequal situation independent of the risk level, because more societal resources are required to attain equality (transferring two “units” rather than one “unit” of health). Others focusing only on relative inequality would be indifferent with respect to these two choices, and those more concerned with inequality among those below a certain level of baseline health might find (5, 5, 4, 4) to be a less desirable situation. As above, the choice of the scale of the inequality measure has important implications for evidence and policy on health inequalities.

### 3.3. Ordinal *versus* Nominal Social Groups

Another potential criterion for choosing a measure of health inequality is the type of the social groups under consideration [29]. Irrespective of their health or exposure status, some social groups have an inherent ordering (“ordinal” groups). For example, there is a clear ordering of social groups defined by income or education, indicators often used to measure socioeconomic position. On the other hand, nominal social groups such as race/ethnicity, or geographic areas, do not have any inherent ordering, though they may obviously be ranked by health or exposure status. Investigating ordinal social groups allows for the quantification of health gradients, meaning situations where measures of health or exposure status either increase or decrease with increasing social group status. Distinguishing ordinal from nominal social groups is useful because certain inequality measures are able to reflect either positive or negative social gradients. 

In some cases one could, for example, create an ordinal-type measure using nominal characteristics across geographic units. For example, by ordering neighborhoods or census tracts by the proportion of minority population, it becomes possible to utilize measures of inequality designed for ordinal comparisons. However, it should be noted that doing so makes an important assumption that the ranking of areas by proportion of minority population is unambiguously associated with increasing disadvantage. Such an assumption may not be tenable if, for example, there are well-off areas with large proportions of minority populations. In addition, assigning the same value to all residents of the neighborhood may mask important within-neighborhood patterns for analyses with geographically resolved exposure information. Such assumptions could be tested or overcome in cases where individual-level data are available on both exposures and social status. But keeping such assumptions and data limitations in mind in the context of group-level data is crucial for a thorough and detailed analysis of inequality.

### 3.4. Explicit Value Judgments

Any inequality measure involves an implicit or explicit weighting scheme that considers transfers/changes in some parts of the distribution more or less significant than transfers/changes in other parts of the distribution. Even those measures without explicit weights involve an implicit decision about weights (*i.e.*, that all populations should be considered identically, rather than considering high-risk or low-risk individuals differently). Because such decisions are inevitable in the context of measuring inequality [2,28], the choice of a specific inequality measure may be more readily made in the context of explicit value judgments about the significance of changes across different parts of health or exposure distributions. 

One way of addressing this concern is to use multiple inequality measures deemed suitable, and to determine if the policy choices are sensitive to the measure selected. Some inequality measures have an explicit weighting parameter, where the value of this parameter influences the relative weights across the distribution. Typically, these parameters can be considered as reflecting the degree of societal aversion toward inequality, or more formally, the amount of weight placed on differences at various points in the distribution [33]. The advantage of these measures is that preferences can be explicitly and quantitatively expressed, and policy choices can be evaluated with respect to the weighting parameter. Even if all decision makers considering environmental justice have some degree of concern about inequality, there may not be consensus regarding how much more weight to place on a change in health risk at the 95th percentile of health risk relative to the median. In general, it is important for any analyst using inequality measures to carefully describe how the measure treats transfers in different parts of the distribution. 

### 3.5. Key Inequality Measures and Their Attributes

Numerous inequality measures have been developed and applied to characterize inequality in health, income, or other attributes. Harper and Lynch [33] listed and described the attributes of 22 inequality measures, and Levy *et al.* [1] identified 19 inequality measures in a literature search and focused on five that were most commonly used or discussed. In Table 1 below, we present a modified version of a table generated by Harper and Lynch [33], focusing on a subset of 20 measures that include some dimension of social group inequality. We characterize these measures by their reference point for comparisons, whether they reflect absolute or relative inequality, whether they have an explicit parameter for inequality aversion, and whether they involve social groups that are ordered (*i.e.*, ordinal) *vs*. unordered (*i.e.*, nominal). Description of all of these candidate inequality measures is beyond the scope of this paper, but in the following section of the paper we define a subset of them (in bold text) that have differing interpretations and offer both within-group and between-group variability, as discussed below.

**Table 1:** Candidate inequality measures and their key attributes. Derived from Harper and Lynch [33].

Inequality measure	Reference group	Absolute or relative inequality	Explicit inequality aversion parameter	Ordered social groups
Absolute Difference	Best off	Absolute	No	Yes
Relative Difference	Best off	Relative	No	Yes
Regression-Based Relative Effect	Best off	Relative	No	Yes
Regression-Based Absolute Effect	Best off	Absolute	No	Yes
Slope Index of Inequality	Average	Absolute	No	Yes
Relative Index of Inequality	Average	Relative	No	Yes
Index of Disparity	Best off	Relative	No	No
Population Attributable Risk	Best off	Absolute	No	No
Population Attributable Risk%	Best off	Relative	No	No
Index of Dissimilarity	Average	Absolute	No	No
Index of Dissimilarity%	Average	Relative	No	No
**Relative Concentration Index**	Average	Relative	Yes	Yes
**Absolute Concentration Index**	Average	Absolute	Yes	Yes
**Between-Group Variance**	Average	Absolute	No	No
Squared Coefficient of Variation	Average	Relative	No	No
**Atkinson Index**	Average	Relative	Yes	No
Gini Coefficient	Average/All those better off	Relative	No	No
**Theil Index**	Average	Relative	No	No
**Mean Log Deviation**	Average	Relative	No	No
Variance of Logarithms	Average	Relative	No	No

## 4. Selecting Health Inequality Measures for Environmental Justice Analyses

As discussed above, between-group comparisons are fundamental to being able to interpret measures of health inequality in the context of environmental justice. Such comparisons can be conducted using straightforward comparisons of distributions between population groups, both before and after a potential policy change. This could involve simple statistical comparisons of mean levels of pollutants or health outcomes, or the fraction of the population above a certain threshold of exposure or risk (e.g., exceeding the 95th percentile of a distribution). While these simple comparisons have the benefit of generally being more transparent and familiar to analysts and policy makers, they have some serious limitations that should be recognized and considered in the context of regulatory analysis. Pairwise comparisons are of diminishing utility as the number of groups, outcomes, or comparisons increases, particularly in the context of evaluating counterfactual exposure scenarios. With a large number of pairwise comparisons, the information may be difficult to present in a straightforward manner, and decision makers may not be able to readily answer the overarching question about whether or not a policy decision will affect broadly defined health inequalities. And in some cases policy decisions may identify social inequalities in health, broadly defined, as the outcome of interest. For example, US policy targets for health inequalities are framed in overall terms (*i.e.*, reducing racial inequalities in health) rather than in terms of specific social group comparisons (e.g., African Americans) [42,43]. Moreover, simple summary metrics may also lead analysts to rely on arbitrary classifications of the population into a small number of groups of interest (e.g., >50% non-Hispanic black, >50% minority) to classify units into exposure categories, which may be unlikely to capture meaningful differences in risk across such thresholds. Finally, the use of simple summary metrics rather than measures that account for the full range of social group distributions may lead to very different conclusions about the magnitude of baseline inequalities, trends over time, or the potential impact of policy changes on inequalities [44]. 

We therefore focus on quantitative measures of inequality that can provide insight about between-group differences while also characterizing overall (or within-group) inequality. Because overall health inequality and social inequalities in health may measure different aspects of distributions of health [45,46], it is useful to explore measures that may quantify each of these components. In particular, we focus on measures of inequality that are additively decomposable, defined as those that can be expressed as the sum of: (1) the inequality between groups; and (2) a weighted sum of inequality within groups [28,30,47]. The main benefit of decomposing inequality into constituent parts is that it can shed light on whether most of the health inequality in a population may be explained by differences in health across social groups [46,48]. This can help to contextualize between-group inequality and can potentially direct analysts toward the groups that have the greatest inequality in exposures or risks. It may also reveal different determinants of the overall distribution of health *vs.* social group differences in health, including aspects of sub-group susceptibility that are important for risk assessment [1]. Moreover, it may be possible that changes over time in policy could affect between-group inequality and have very little impact on overall inequality, or vice versa. The primary advantage of using additively decomposable inequality measures is that it allows one to determine not just whether between-group inequality is increasing, but whether the share of total inequality that is due to inequality between groups is increasing or decreasing. In any case, both dimensions of inequality should be kept in mind in the context of any analysis of inequality change.

### 4.1. Selected Decomposable Inequality Measures

Below we describe in detail some selected measures of inequality that may be used to measure both overall and between-group inequality. More exhaustive reviews and technical details of other measures can be found elsewhere [28,30,49].

#### 4.1.1. Variance

Multiple inequality measures in Table 1 are derived from the Variance. For example, one can take the logarithm of the health/risk measure, in which case it is called the Variance of Logarithms (*VarLog*), or one can normalize the health/risk measure by the mean, in which case it is called the squared Coefficient of Variation (*CV*^2^). The Variance is also widely recognized and easy to communicate to decision makers and others familiar with basic statistics. We therefore briefly discuss key aspects of the Variance. 

The generic formula for the total variance of a distribution is:

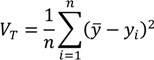
(1)
where *y_i_* is a measure of health/exposure status for individual *i*, *y* is the mean health/exposure of the population, and *n* is the number of individuals in the population. The Variance is therefore a measure of absolute inequality with the average member of the population as the reference point for comparisons. 

The Variance can also be easily decomposed into between-group and within-group components. For a simple two-group decomposition (e.g., for rich and poor), the total variance can be written as a function of two parts. The between-group part is calculated by assigning rich and poor individuals the average health of their respective groups, and taking the variance of that distribution of groups (this is essentially equivalent to what the variance would be were there no inequality within social groups); and the within-group part is calculated by calculating the variance separately for rich and poor and taking a weighted average of those two variances, with the weights equal to the share of total observations in each group [28,30]:

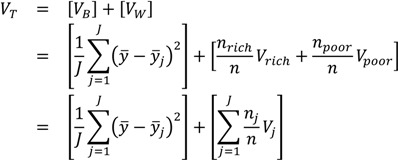
(2)
where *V_B_* and *V_W_* are, respectively, the between-group and within-group variance, *y_j_* is the mean health of the *j*th group, *V_rich_* and *V_poor_* are, respectively, the variance estimated separately among the rich and among the poor, and *n_rich_* and *n_poor_* are the numbers of rich and poor individuals in the population. The third equation simply shows that the within-group inequality component may be extended to include *J* groups, as most environmental justice analyses will consider more than one population group (e.g., multiple racial/ethnic groups, multiple income strata, multiple geographic areas).

In the context of analyses focused only on estimating the magnitude of inequality between groups, the first bracketed term on the right-hand side of Equation (2) may be used to measure inequality between groups and is sometimes called the Between Group Variance [33]. Because it is applicable in the context of any social group comparisons, the Between Group Variance may be a useful indicator of absolute inequality for social groups that do not have any inherent ordering (e.g., across geographic units, or across racial/ethnic groups). 

The Variance does not have an explicit inequality aversion parameter, but it does incorporate an implicit weighting by squaring differences and therefore placing a greater weight on large differences from the average. Thus, any decision maker using this measure should be comfortable with the interpretation that a large difference for a small number of people could outweigh a small difference for a large number of people. 

Some of the modified forms of the Variance, such as *VarLog* and *CV^2^*, share similar attributes as the Variance but function as measures of relative inequality. Both *VarLog* and *CV*^2^ are also additively decomposable inequality measures, but require adjustments to the weighting scheme for the within-group inequality component [1,28,30]. 

#### 4.1.2. Measures of Entropy: Theil Index and Mean Log Deviation

Measures of general entropy may also be used in the context of measuring within- and between-group inequality in health [50]. This family of measures may be less familiar to decision makers and regulatory analysts than the family of measures derived from the Variance, but they offer some significant advantages. Generalized measures of entropy incorporate a parameter that allows for differential sensitivity of the resulting index to different parts of the health distribution [30,49]. The parameter value leads to the choice of a specific index within the family of measures of general entropy. Two common indices of inequality that are part of the class of entropy-based measures are the Theil index (*T*) and the Mean Log Deviation (*MLD*) [51]. For individual-level data, total inequality in health/exposure *y* measured by the Theil index can be written [52] as:

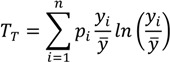
(3)
where *p_i_* is an individual or group’s population share (which in the case of individual data will be 1/*n*, so that ∑ *p_i_*=1) and *y_i_/y* is the ratio of the individual or group *i*'s health to the average health of the population. When the population of individuals is arranged into *J* groups, the equation is the exact sum of two parts: between-group inequality and a weighted average of within-group inequality:

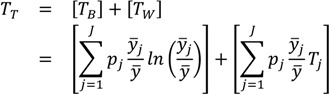
(4)
where *T_B_* is the between-group Theil index, *y_i_* is the average health in group *j*, *T_W_* is the total within-group Theil index, and *T_j_* is the inequality in health within group *j*. The within-group component [the second term on the right side of Equation (4)] is effectively weighted by group *j*’s share of total health, since *p_j_* × *y_i_/y* = *y_j_* (where *y_j_* is the share of total health in group *j*). The above decomposition of *T_T_* also makes it clear that it is possible to calculate between-group inequalities in health without having data on each individual's health status. The only data needed are the proportions of the population in each social group (*p_j_*) and the ratio of the group’s health to that of total population (*y_i_/y*). Given the structure of most regulatory analyses and the reliance on Census data that are characterized at spatially aggregate levels, this is an appealing feature. 

As with many other common inequality measures, the Theil index involves a comparison with the average and is a relative rather than an absolute measure (Table 1). Using the Theil index involves a choice among the various generalized measures of entropy. Thus, an explicit inequality aversion parameter is involved in the selection process, with the statistical formulation of the Theil index placing greater weight at the upper end of the distribution. However, there is no explicit inequality aversion parameter within the Theil index itself, so one cannot characterize differential sensitivity to inequality without also considering other measures (within the generalized entropy family or otherwise). While this measure has very attractive qualities, the between-group/within-group decomposition requires continuous outcome data estimated for individuals, so it is not clear whether this can be applied for some binary health outcomes (e.g., incidence, mortality or screening). But even for non-continuous outcomes entropy indices can easily be used to calculate between-group inequality in the absence of individual-level data. For example, suppose that absent a continuous indicator of risk we wanted to measure the between-group disparity in cancer mortality rates. This could be accomplished by calculating the first term on the right side of the above equation (*T_B_*) using the data on each group’s proportion in the population (*p_j_*) and their rate of mortality relative to the overall population rate (*y_i_/y*)—data that may be readily available.

Another entropy-based measure that is additively decomposable is the Mean Log Deviation (*MLD*), sometimes called Theil’s second measure. One way [52] of writing the formula for the total *MLD* is:

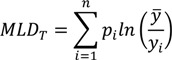
(5)
where the quantities *p_i_* and *y* are defined as above for the Theil index. To decompose *MLD_T_* into between-group and within-group components the following formula [52] may be used:

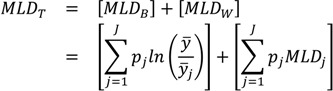
(6)
where *MLD_B_* is the between-group *MLD*, *MLD_W_* is the total within-group *MLD*, and *MLD_j_* is the inequality in health within group *j*. Again, it is straightforward to see that the total within-group component is a weighted average of the within-group inequalities, with weights equal to the population size of each social group. 

The main difference between *T* and *MLD* is differential sensitivity to different parts of the health distribution, with the former being more sensitive to the upper part of the health distribution, and the latter the lower part of the distribution. Additionally, *T* is weighted by shares of health in each social group, whereas *MLD* is weighted by shares of population. Thus, in the context of regulatory analyses, the *T* will be somewhat more influenced by groups with larger health ratios (*y_i_/y*), whereas *MLD* will be somewhat more influenced by groups with large population shares (*p_j_*). It should also be noted that a ‘symmetrized’ entropy index has been proposed [53] to measure between-group inequality that is effectively a weighted average of the *T* and *MLD*.

#### 4.1.3. Atkinson Index

The Atkinson index has been used in a number of income and health inequality applications, in part because it has many desirable features, including sub-group decomposability and an explicit inequality aversion parameter. The overall index may be written as:

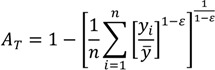
(7)
where *y_i_* represents the health status of the *i*th individual, *n* represents the number of individuals, and ε represents an inequality aversion parameter. 

In contrast to the class of general entropy measures above, the Atkinson index is not strictly additively decomposable. However, it may be usefully decomposed into a between-group component, a within-group component, and a residual term that is minus the product of the between and within components [1,30]. By replacing each individual’s health/exposure with the average of the value for their social group, one can use the Atkinson index to measure between-group inequality:

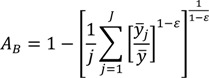
(8)
where now *y_j_* represents the average health of group *j*. The formula for the within-group component is somewhat more complicated than for the entropy-based measures above, and is given by Cowell [49]. 

The explicit inequality aversion parameter is an appealing feature of the Atkinson index in the context of an environmental regulatory analysis where it is important to make transparent any assumptions about how different populations have been weighted. However, one concern that has been raised is the fact that an increase in the inequality aversion parameter places increasing weight at the bottom of the distribution, whereas one would prefer increasing weight at the top of the distribution if characterizing adverse health outcomes. This can be addressed by characterizing health as a “good” when theoretically appropriate, by applying basic transformations to the health measure (*i.e*., using the inverse of risk), or by working with a narrow range of inequality aversion parameters that are empirically justified and avoid more extreme interpretations.

#### 4.1.4. Concentration Index

The Concentration Index (*CI*) has been used extensively in the health inequality literature. It involves ordering the population first based on an ordinal social grouping, and then plotting the cumulative percentage of the population against a cumulative measure of health [33]. It therefore can be displayed graphically, which could help decision makers to better understand and interpret the results. The *CI* can either be relative, if calculated as a percent of the total amount of health, or absolute, if calculated as the cumulative amount of health. It can also be defined with respect to a positive health status measure or an indicator of adverse health outcomes, and can be constructed for defined social groups or at an individual level. The *CI* has a number of statistical formulations, but a common version [20] of the relative *CI* is:

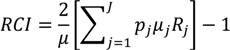
(9)
where *p_j_* is the social group’s population share, *μ_j_* is the group’s mean health, and *R_j_* is the relative rank of the *j*th socioeconomic group, which is defined as:

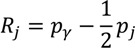
(10)
where *p_γ_* is the cumulative share of the population up to and including group *j* and *p_j_* is the share of the population in group *j*. *R_j_* essentially indicates the cumulative share of the population up to the midpoint of each group interval. The absolute Concentration Index (*ACI*) is obtained by multiplying the *RCI* by average health [33].

While the formula for the *RCI* above does not have an explicit parameter for inequality aversion, there is an extended version of the *RCI* that offers this capability [21,54]. The aversion parameter changes the weight attached to the health of different socioeconomic groups in a manner similar to the Atkinson index described above. The formula for this extended version of the *RCI* for grouped data is:

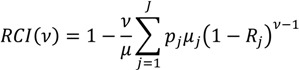
(11)
where *ν* is the “aversion parameter” and the other quantities are defined as in Equation (9) above. Generally, the weight attached to the health of lower socioeconomic groups increases and the weight attached to the health of higher socioeconomic groups decreases as ν increases. For the “standard” *RCI* the value of the parameter (*ν*) is 2, which leads to respective weights of 2, 1.5, 1, 0.5, and 0 for the health of individuals at the 0th, 25th, 50th, 75th, and 100th percentile of the cumulative distribution according to socioeconomic position [21]. The health of the poorest person in the population is thus weighted by 2 and the weights decline as socioeconomic rank increases. Since this inequality aversion parameter may be adjusted, decision makers could potentially specify exactly how much weight to give to each social group in the context of a regulatory analysis. It should also be noted that the issue of sensitivity of the *RCI* to how binary health indicators are considered (presence *vs.* absence of disease remains a concern for incorporating the aversion parameter [54]. 

The *CI* offers population group decomposition, although not in a strictly additive sense [55]—it is equal to the sum of a between-group component (comparing the mean levels of health across population groups), a within-group component (a weighted sum of the population group concentration indices, where the weights are the product of the health share and the population share), and a residual term that is present if the population groups overlap in their income ranges or other social group ordering. However, it requires an explicit social group ordering, so it may not be suitable for situations in which there are not obvious rankings of social groups. Income or other measures of socioeconomic status have a strict ordering, but race/ethnicity and other demographic characteristics do not. As noted above, it may be possible to rank geographic areas with respect to nominal characteristics, though doing so involves making assumptions that should be carefully considered. 

### 4.2. Selection and Application of Inequality Indicators for Environmental Justice Analyses

We would consider as plausible candidate indicators to incorporate environmental justice into regulatory analyses any quantitative measures that adhere to basic rules for inequality indicators, and that allow for decomposition of inequality into between-group and within-group components. All of the indicators listed in Section 4.1 meet those criteria, as do others in the literature. While a number of questions and factors could be considered before arriving at the subset of candidate indicators, we consider three to be most significant: (1) Are relative or absolute measures of inequality of greater concern?; (2) Is there a desire to have an explicit inequality aversion parameter within a single selected indicator?; and (3) Are the population groups to be compared in the environmental justice analysis inherently ordered (e.g., an income gradient)?

The first two of these questions are policy decisions to be made at the level of regulatory decision makers. As discussed above, there are compelling arguments to be made for both relative and absolute concepts of health inequality. Decision makers could conclude that one construct is more suitable given their understanding of environmental justice, or could determine that either concept is reasonable and evaluate the sensitivity of policy conclusions to this choice. The desire for an explicit inequality aversion parameter would be a preference that decision makers might express given the objective to minimize implicit policy decisions within the inequality indicator calculations, although this same preference could be met by using multiple alternative indicators (e.g., multiple generalized entropy measures). The third question may be influenced by the application and subset of environmental justice questions under consideration – socioeconomic status may be the more pertinent measure for some policies, while race/ethnicity may be the more pertinent measure for others. 

After choosing a decomposable measure of inequality and assembling the requisite health/exposure data, analysis and decomposition of inequalities is relatively straightforward. All of the equations listed above can be readily implemented in spreadsheets or statistical analysis software [56,57,58]. Measures of uncertainty exist for most inequality measures (or may be estimated using bootstrapping or other resampling techniques) [56,57,59], and should also be reported alongside point estimates. However, appropriate interpretation of point estimates may be less straightforward. In a regulatory analysis context, the crucial questions will often involve comparing inequality measures before and after proposed policy measures. In situations where only a single policy option is considered, it is challenging to determine whether the magnitude of any change in inequality (positive or negative) is important relative to other decision-relevant metrics, though the direction of changes is clearly interpretable. For regulatory analyses in which multiple options are under consideration, the changes in inequality measures can be used along with other metrics to determine policy options that best meet multi-attribute decision criteria. For example, one analysis building on an EPA case study [19] compared two emissions control strategies and showed that a multi-pollutant/risk-based approach both led to greater health benefits and more reductions in health inequality (as measured by the Atkinson index and Gini coefficient). This analysis also showed that overall inequality was dominated by differences in risk between vulnerable/susceptible individuals and the rest of the population, reinforcing an environmental justice framework. Another study examined many hypothetical emission control strategies for power plants and determined the subset of policies that were optimal with respect to both total health benefits and reductions in health inequality [17].

At times, regulatory analyses (or environmental justice analyses in other contexts) will involve examining baseline trends over time to determine whether circumstances have been improving or getting worse. While this analysis is computationally straightforward, interpreting changes over time in between-group inequality may be complicated, especially over longer periods. One potential complication is that changes in relative and absolute inequality may diverge, leading to potentially opposing conclusions about the effect of the policy on health inequalities. A second complication occurs because changes in the value of between-group inequality are a function of two quantities: changing social group proportions and changing health status among social groups. Differentiating between these two components of change may be important from an environmental justice perspective. If between-group inequality is increasing but the main reason for the observed change is that the share of the population among groups at the tails of the health distribution has increased, it simply demonstrates that the inequality increase is primarily due to the movement into and out-of different social groups and may not be the result of differential changes in health within those groups. This explanation would not necessarily imply that between-group inequality would not be a growing concern, but would emphasize that demographic patterns and other societal factors explain the trends better than changing environmental exposures. On the other hand, if we find that population shares have remained relatively constant over time (likely in the case of shorter periods of observation) but between-group inequality has increased because of changes in the health status of social groups, this implicates differential sources of changes in health status and may imply a need to address the causes of differential health change.

## 5. Summary and Conclusions

In this paper, we have provided both theoretical and empirical arguments that measurement of health inequality is feasible in the context of environmental justice analyses conducted for evaluating regulatory policy. Health inequality has been characterized in numerous prior investigations following well-established approaches. The questions from the perspective of environmental justice relate to the context in which an inequality measure would be used, the data required for a meaningful measure of health inequality, the criteria for selecting inequality measures to apply in regulatory analyses, and the ultimate application and interpretability of the results. 

The regulatory analysis application implies an orientation around health outcomes and how they are distributed, both at present and after a potential policy change. In addition, conceptions of environmental justice suggest that pre-defined social groups are to be given direct consideration. These two contexts emphasize how inequality measures should be applied within regulatory analyses—with characterization of both baseline inequality and how inequality would change given a policy change, and utilizing between-group comparisons while also considering within-group inequality. To make this characterization meaningful, the health risk models must have sufficient resolution to allow for between-group and within-group variation in exposure and susceptibility. The geographic resolution would need to be consistent with both available demographic data and the anticipated spatial contrasts of the exposure—the resolution required to characterize a near-roadway environment would be different from the resolution required for regional air pollution. Ideally, data on differential baseline disease rates or effect modification by demographics relevant to environmental justice analyses would also be available. Absent information of this sort, even the most theoretically desirable inequality measure will not yield meaningful insights. That said, most regulatory analyses involve spatially resolved characterizations of exposure and/or health risk, at baseline and after proposed regulations. So, the analytical foundation is generally in place for health inequality assessments, with the need to ensure that sociodemographic information is considered wherever possible. 

Given sufficient information to characterize health risks at baseline and after proposed regulations, decision makers and analysts must choose among candidate inequality measures. In this paper, we do not recommend a specific inequality measure, largely because this is a policy choice. However, we do outline the questions that decision makers would need to ask and answer in order to focus on the subset of indicators best representing their values. Specifically, a decision is necessary regarding whether relative or absolute concepts of inequality are more appropriate; whether a selected indicator must have an explicit inequality aversion parameter (and, if so, the degree of aversion); and whether any environmental justice analyses would involve comparisons only among inherently ordered population groups (*i.e*., socioeconomic gradients). These are not simple questions to answer, and it is likely that multiple views exist on these questions. Therefore a default to a suite of inequality measures representing a range of viewpoints would seem a reasonable choice. In some situations in which multiple policy options were under consideration, a single option will emerge as preferable across all candidate inequality measures. In this case, the choice among policy options is clear, at least from an environmental justice perspective. When the choice among policy options differs across inequality measures, analysts will need to articulate the basis for this difference and in particular, lay out the concepts of inequality that will inform the choice of one policy over another. If decision makers chose a default inequality measure and perspective on inequality, this decision would clarify the implications of that choice. If no default were developed, differences across inequality measures could mean that there is no ideal measure with respect to environmental justice (in which case policy choices could be based on other criteria), or that new policy options could be developed to better reduce exposures among high-risk population groups and therefore more clearly improve environmental justice. 

Quantitative measures of inequality cannot represent all dimensions of environmental justice, and analysts should be clear about this point. That said, inequality measures provide important insight into how patterns of health risks are changing over time and space, and if selected and presented appropriately, can make meaningful contributions to regulatory analyses of environmental justice.
